# TWN-FS method: A novel fragment screening method for drug discovery

**DOI:** 10.1016/j.csbj.2023.09.037

**Published:** 2023-09-29

**Authors:** Hye Ree Yoon, Gyoung Jin Park, Anand Balupuri, Nam Sook Kang

**Affiliations:** Graduate School of New Drug Discovery and Development, Chungnam National University, 99 Daehak-ro, Yuseong-gu, Daejeon 34134, South Korea

**Keywords:** Water network, Hot spots, Fragment screening, Fragment-based drug design, CDK2

## Abstract

Fragment-based drug discovery (FBDD) is a well-established and effective method for generating diverse and novel hits in drug design. Kinases are suitable targets for FBDD due to their well-defined structure. Water molecules contribute to structure and function of proteins and also influence the environment within the binding pocket. Water molecules form a variety of hydrogen-bonded cyclic water-ring networks, collectively known as topological water networks (TWNs). Analyzing the TWNs in protein binding sites can provide valuable insights into potential locations and shapes for fragments within the binding site. Here, we introduce TWN-based fragment screening (TWN-FS) method, a novel screening method that suggests fragments through grouped TWN analysis within the protein binding site. We used this method to screen known CDK2, CHK1, IGF1R and ERBB4 inhibitors. Our findings suggest that TWN-FS method has the potential to effectively screen fragments. The TWN-FS method package is available on GitHub at https://github.com/pkj0421/TWN-FS.

## Graphical Abstract

**Figure:**
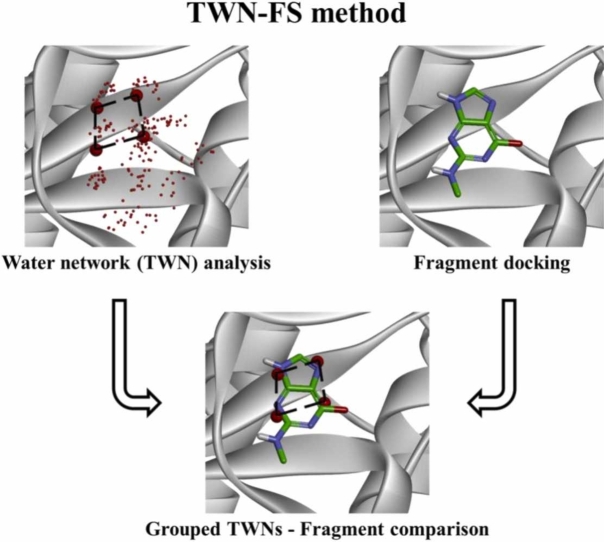

## 1 Introduction

Fragment-based drug design (FBDD) has emerged as a highly effective method for early-stage drug discovery and development over the past two decades. This method offers several notable advantages including enhanced hit rates through the utilization of smaller screening libraries and the consideration of improved physicochemical properties [1], [2], [3], [4], [5]. The FBDD strategy involves identifying fragments that interact with the binding hot spot [6], which refers to the specific region in the binding site contributing the most to the protein-ligand binding free energy. Subsequently, these fragments are linked, grown and merged to design potential drug candidates [7], [8]. Kinases are important targets for the drug development and FBDD is particularly suitable for the development of novel kinase inhibitors because the binding pockets of kinases are well-characterized. Specifically, the hinge region (known as the Adenine pocket or AP site) serves as a significant binding hot spot in the kinase structure [9]. This region connects the N-terminal and C-terminal, and plays a crucial role in the interaction with kinase type 1 inhibitors [10]. Consequently, in the design of kinase type 1 inhibitors, the scaffold is designed to mimic the adenosine moiety [11], facilitating its interaction with the hinge region, or the scaffold hopping method [12] is employed to explore diverse scaffolds that maintain essential interactions with the hinge region.

Water molecules surrounding the protein surface play a crucial role in mediating protein folding, maintaining structure, enabling proper function and facilitating ligand binding [13]. Recognizing their significance, several research groups have explored the importance of water molecules in the field of drug discovery. Through various case studies, researchers have demonstrated that predicting the positions of water molecules and understanding hydration sites can enhance protein-ligand binding affinity [14], [15] or identify regions where fragments can be substituted [16], [17]. The studies on water molecules primarily focus on free energy calculations. Various algorithms are used for these calculations including single static structure-based approaches and simulation-based approaches. Single static structure-based methods like such as SZMAP [18] and WaterFLAP [19] employ water probes to identify potential binding sites and determine hydration sites. While these methods offer the advantage of not requiring prolonged equilibration and extensive sampling to obtain reliable results, a drawback is that they tend to overlook the dynamic and cooperative aspects that govern hydration. There are instances where atom substitutions have been carried out using water networks based on WaterFLAP without considering water cooperative effects [20]. This approach has the drawback of requiring MD simulations on the protein-ligand complex. On the other hand, simulation-based methods such as WaterMap [21], [22], STOW [23], and MobyWat [24], [25] or HydroDock [26] utilize explicit water molecules that can freely explore space of the system. These methods calculate the binding free energy and thermodynamic properties including the position, occupancy, enthalpy and entropy of water molecules at the ligand binding site. In MobyWat or HydroDock, the approach involves connecting solvent positions by applying a distance threshold of 3.5 Å. As a result, the generated networks extend significantly across the entire binding region. However, when opting for a position-based network analysis with MobyWat, an additional energy calculation step becomes necessary. This requirement arises due to the inherent limitation of position-based networks which lack information pertaining to occupancies and cooperative behavior that are essential for linking network connectivity to solvation energetics. Furthermore, WaterMap and STOW methods are technically challenging, computationally demanding, time-consuming as they require multiple simulations to account for individual water molecules. Moreover, they have limitations in understanding the overall stability of hydrogen-bonded networks formed by water molecules in protein binding sites. Furthermore, the removal of a single water molecule can influence the free energies and structure of the remaining water network. Free energy-based methods require additional simulations to account for these changes. Additionally, the individual binding free energy of each water molecule does not provide insight into cooperative effects between neighboring water molecules without further analysis.

Previously, we introduced a novel approach called topological water network (TWN) analysis. This method enables the identification of water networks without the need for computationally expensive explicit free energy calculations and considers the cooperative effects among adjacent water molecules. We effectively applied this method for various applications including designing experimentally validated kinase inhibitors [27], [28], [29], studying kinase selectivity [30], analyzing protein-ligand binding [27], exploring drug repurposing [31], [32], investigating protein hydration [33] and gaining insights into protein folding [34]. Furthermore, we introduced a TWN-based residue encoding (TWN-RENCOD) method for comparing protein binding sites that takes into account the distances between TWNs and binding site residues to calculate the similarity between protein binding sites [35].

The objective of this study is to investigate the applicability of TWNs as a FBDD screening method, with the underlying hypothesis that locations of TWNs within the protein binding site are indicative of hot spots and can be substituted by fragments with similar sizes and shapes. Here, we propose the TWN-fragment screening (TWN-FS) method, a novel grouped TWNs based fragment screening method. The grouped TWNs can provide valuable insights into the geometry and shape requirements for substituting fragments in the protein binding hot spots without the energy calculations. Unlike our previous works, we have focused on the 4-membered ring TWN in this method instead of the 3-membered ring TWN. We opted for the 4-membered ring TWN due to its lower occurrence in the protein binding site compared to the 3-membered ring TWN. Additionally, the 4-membered ring TWN demonstrates a size comparable to fragments and the majority of its conformations exhibit a planar structure which facilitates the calculation of orthogonal distances to fragments. In contrast to our previous TWN-based approaches, instead of analyzing all the molecular dynamics (MD) trajectories individually, we merged all the trajectories and conducted the analysis collectively on the combined trajectories in the TWN-FS method. TWNs appearing frequently in the protein binding site were observed to have a high density in the merged MD trajectories. Through this observation, we hypothesized that specific locations in the binding site which exhibit high density of TWNs could serve as potential sites for replacing TWNs with fragments. Accordingly, we identified and grouped the densely populated TWN locations in the protein binding site, with the goal of proposing fragments of similar size to TWNs that could be potentially substituted at these locations.

The protein hot spot location was assessed individually for 27 kinases using KinfragLib [36], which is a database containing crystallographic ligand fragments. In the kinase selection process, we opted for 25 kinases with the high count of AP fragments in KinFragLib. Additionally, we included one kinase with a moderate count of AP fragments and another kinase with a low count of AP fragments. For selecting fragments, we calculated shape similarity and average distance values between grouped TWNs and fragments. We established threshold values for shape similarity and average distance based on the grouped TWNs observed in the binding sites of several kinases and all known crystallographic kinase fragments. In order to investigate the efficacy of our TWN-FS method, we screened known CDK2, CHK1, IGF1R and ERBB4 inhibitors from the ChEMBL database using this method specifically focusing on the AP site of each protein. This allowed us to assess the effectiveness of our approach in identifying potential hits.

## 2 Material and methods

### 2.1 TWN-FS workflow

**Fig. 1:**
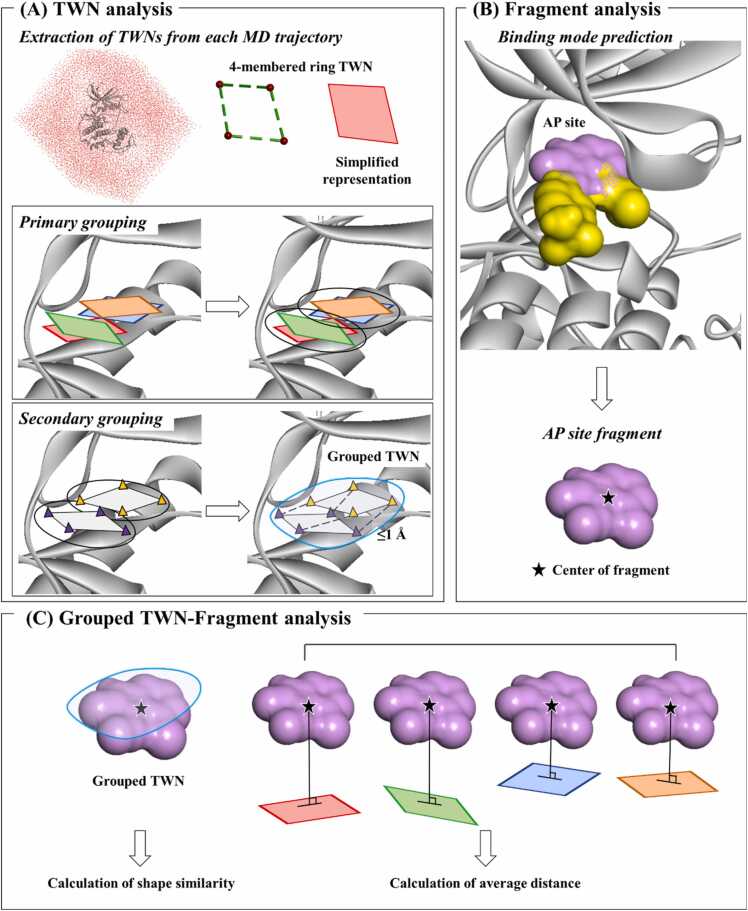
The overview of TWN-FS methodology. (A) TWN analysis: This process involves MD simulation, followed by the extraction and analysis of the 4-membered ring TWNs observed within the protein's binding site. The primary grouping clusters nearby TWNs in specific areas (black circles). The secondary grouping is obtained by comparing the primary groupings with each other, considering that the center of each O atom cluster (purple and yellow triangles) in the primary grouping is less than or equal to 1 Å (blue circle). The grouped TWNs are finally formed after two sequential steps of grouping. (B) Fragment analysis: In this process, fragments are initially docked into the protein binding site and fragments located at a specific site (e.g. AP site) are selected. The AP site fragment is represented by a pink solvent surface and its centroid is marked with a black star. (C) Grouped TWN–Fragment analysis: This process involved calculation of the shape similarity and average distance between the final grouped TWNs (Fig. 1A) and fragments (Fig. 1B). Finally, fragments meeting specified threshold criteria for shape similarity and average distance values are selected.

In the TWN-FS method (Fig. 1), initially the co-crystallized ligand is extracted from the X-ray crystal structure of the target protein and then MD simulation is performed on the protein structure in the absence of the ligand. The co-crystallized ligand is excluded in order to focus on analyzing the formation of the water networks within the binding site of the protein. During the simulation, the protein is restrained and only the water molecules are permitted to move. Subsequently, TWNs are analyzed in all MD trajectories. The planar as well as non-planar 4-membered ring TWNs observed within the protein binding site are extracted for further investigation. This method is based on the hypothesis that regions with high density of TWNs could potentially be replaced with fragments, specifically fragments that closely match the size of the TWNs. Accordingly, 4-membered ring TWNs with high density within the specified region are grouped and analyzed. The grouping process was carried out in two sequential steps, namely primary grouping and secondary grouping.

The primary grouping focused on clustering TWNs that were spatially close to each other within the specified region. Conversely, the secondary grouping aimed to group these primary clusters into expanded regions. The final grouped TWNs resulting from these grouping steps are used for fragment screening. In the fragment screening process, fragments are firstly docked in the binding site of the target protein to predict their binding modes. Afterwards, the shape similarity and average distance between the grouped TWNs and fragments are calculated. Finally, fragments whose shape similarity and average distance values meet the specified threshold are selected.

### 2.2 Protein preparation

The kinase–ligand interaction fingerprints and structure database (KLIFS) database [37] contains a consistent structural alignment of the kinase domains from the numerous Protein Data Bank (PDB) structures covering different human kinases. The KLIFS structural alignment allows the comparison of all structures and ligands to each other. The X-ray crystal structures of 27 human kinases were retrieved from the KLIFS database. These kinases included CDK2 (PDB 4ERW) [38], CHK1 (PDB 1NVR) [39], p38a (PDB 4F9Y) [40], JAK2 (PDB 4IVA) [41], ERK2 (PDB 4ZZN) [42], CK2A1 (PDB 5T1H), SYK (PDB 1XBC) [43], EGFR (PDB 4ZAU) [44], GSK3B (PDB 1Q3D) [45], AURKA (PDB 5DT0) [46], BTK (PDB 6×3N) [47], BRAF (PDB 3Q4C) [48], DYRK1A (PDB 4NCT) [49], FGFR1 (PDB 5EW8) [50], JNK3 (PDB 4Z9L) [51], LCK (PDB 3AC1), CHK2 (PDB 2CN5) [52], JAK1 (PDB 6N7A) [53], PDK1 (PDB 1OKY) [54], TGFBR1 (PDB 5QIM) [55], MELK (PDB 5M5A) [56], JAK3 (PDB 5LWM) [57], WEE1 (PDB 1×8B) [58], IGF1R (PDB 3NW6) [59], EPHA2 (PDB 5IA2) [60], HCK (PDB 2HK5) [61] and ERBB4 (PDB 2R4B) [62]. The full names of these proteins are provided in Table S1. All non-protein molecules were removed from the structures and subsequently the structures were processed using the ‘Prepare Protein’ module in Discovery Studio 2022 (BIOVIA, San Diego, CA, USA). The protein preparation process included the addition of missing residues and hydrogen atoms, and assignment of bond orders and formal charges. Additionally, protonation states were assigned at a pH of 7.4.

### 2.3 MD simulation

MD simulation was performed using GROMACS version 5.1.4 [63] and CHARMM27 all-atom force field [64]. The co-crystallized ligand was extracted from the X-ray crystal structure of the target protein and then MD simulation was conducted on the protein structure in the absence of the ligand. Protein was solvated in a cubic box using the TIP3P water model [65]. Counter ions were added to ensure the neutrality of the system. Subsequently, the system was subjected to energy minimization using 50,000 steps of the steepest descent algorithm. Equilibration was conducted in two phases. The system was firstly equilibrated for 100 ps in the NVT ensemble (constant number of particles, volume and temperature) at a temperature of 300 K using V-rescale thermostat [66]. Afterwards, the system was equilibrated for 200 ps in the NPT ensemble (constant number of particles, pressure and temperature) at a pressure of 1 bar using the Parrinello–Rahman barostat [67]. Finally, production run was carried out for 10 ns with restraints on the protein. The time step used for the simulation was 1 fs. Periodic boundary conditions (PBC) were used in the MD simulation. A linear constraint solver (LINCS) algorithm [68] was used to constrain bonds. A cut-off of 12 Å was used for short-range electrostatics, while long-range electrostatics were calculated using the Particle mesh Ewald method [69]. For each protein, 1000 MD trajectories were extracted for the TWN analysis.

### 2.4 TWN analysis

**Fig. 2:**
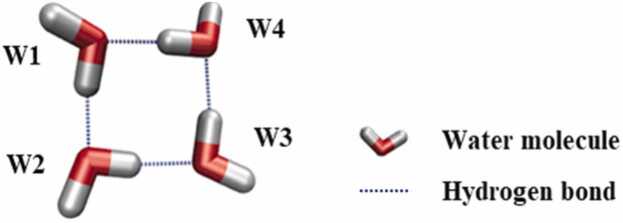
An illustration of a 4-membered ring TWN. Water molecules involved in the 4-membered ring TWN are shown as stick models (W1-W4) whereas hydrogen bond interactions are displayed as dotted lines.

Water molecules form various types of hydrogen bonded cyclic water ring networks known as TWNs. The 4-membered ring TWN represents a specific type of TWNs comprising a cyclic water-ring network formed by four water molecules interconnected through hydrogen bonds (Fig. 2).

The interaction potential energy between water molecules in TWNs is estimated using the Lennard-Jones and Coulomb potentials. These potentials are based on a rigid TIP3P water model. The equation below shows how the interaction potential energy is calculated between two water molecules (*a* and *b*).

$$v\left(a,b\right)=\sum_{i}^{\mathrm{on}\;a}\sum_{j}^{\mathrm{on}\;b}\frac{q_{i}q_{j}e^{2}}{r_{\mathrm{ij}}}+\frac{A}{{r_{\mathrm{oo}}}^{12}}-\frac{C}{{r_{\mathrm{oo}}}^{6}}$$

where:

v(*a*, *b*): interaction potential energy.

r_oo_: distance between oxygen atoms.

q_i_: partial charge on the *i* site (−0.834*e*).

q_j_: partial charge on the *j* site (0.417*e*).

r_ij_: distance between *q*_*i*_ and *q*_*j*_.

*A*: repulsive force between *i* and *j* (582,000 kcal Å^12^ mol^−1^).

*C*: attractive force between *i* and *j* (595 kcal Å^6^ mol^−1^).

The parameters were carefully selected to ensure that they produce realistic structural and energetic properties of liquid water. A threshold energy criterion of − 2.25 kcal mol^−1^ was used to determine the existence of hydrogen bonds between water molecules, as this value corresponds to the minimum value of the pair-energy distribution of potential. The equation provided above was utilized to analyze planar as well as non-planar 4-membered ring TWNs in each extracted MD trajectory. In the TWN analysis, 4-membered ring TWNs within a 20 Å radius around the protein DFG region were extracted. The choice of a 20 Å radius was made to include the protein binding site and ensure that it does not affect the analysis due to the differences in the protein binding sites. Consequently, the extraction was performed with a larger radius than the binding site itself.

### 2.5 TWN grouping

**Fig. 3:**
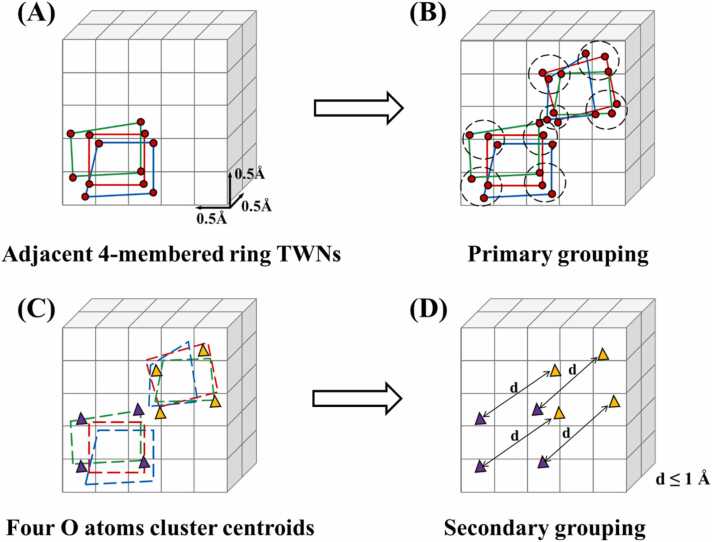
The outline of TWN grouping process. The 4-membered ring TWNs are displayed as green, red and blue lines while oxygen (O) atoms within these TWNs are represented as red spheres. The cluster of each O atom constituting the TWN are indicated by a dotted black circle. The centers of O atom clusters of the adjacent TWNs are displayed as purple and yellow triangles while ‘d′ represents the distance between the centers. (A) Initially, the coordinate information of each O atom within the 4-membered ring TWNs is recorded in a 3D grid with 0.5 Å spacing. (B) Primary grouping clusters all TWNs which are located in close proximity to each other. (C) Subsequently, centers of the O atom clusters are determined. (D) Lastly, secondary grouping compares centers of the O atom clusters and group all TWNs whose centers were within a distance (d) of 1 Å to provide the final grouped TWNs.

The TWNs observed within the protein binding site were extracted from each MD trajectory. Subsequently, 4-membered ring TWNs with high density within the AP site were grouped. The grouping of TWNs was conducted based on the oxygen (O) atoms within each 4-membered ring TWN. This process involved two sequential steps, namely primary grouping and secondary grouping (Fig. 3).

The purpose of the primary grouping was to cluster 4-membered ring TWNs that were spatially close to each other. In this process, the coordinate information of each O atom within the 4-membered ring TWNs was stored in a 3D grid with a spacing of 0.5 Å. The TWNs were grouped by expanding the unit around each O atom by 0.5 Å on each axis, creating four extended zones. Grouping occurred when neighboring TWNs fall within these extended zones. This grouping considered all extracted 4-membered ring TWNs and grouped them if all four O atoms within the extended unit meet the criteria. Primary grouping lead to the clustering of all 4-membered ring TWNs that were located in close proximity to each other and existed at nearly the same location. The secondary grouping was performed based on the results of the primary grouping. This process further refined the grouping of TWNs based on their spatial proximity by combining the primary clusters into larger expanded regions. After the primary grouping, the O atom clusters formed four distinct regions. To determine the center of each cluster, the density-based spatial clustering of applications with noise (DBSCAN) algorithm [70] was employed. The centers of the four O atom clusters were compared, and all the primary grouping TWNs whose centers were within a radius of 1 Å were grouped together to obtain the final grouped TWNs. The number of units, TWNs and grouped TWNs for each protein are provided in Table S1.

### 2.6 Grouped TWNs-fragment analysis

#### 2.6.1 Grouped TWNs-fragment shape similarity calculation

The shape similarity calculation between the fragment and the grouped TWN uses the reminiscent of Shape and Electrostatic Potential (ShaEP) method [71] and is carried out without superimposition to preserve the location information of the TWN. Only the shape similarity value obtained from the ShaEP method is used as the electrostatic potential value is not applied to the TWN. The shape-density of atom *i* at ***r*** is expressed as a spherical Gaussian

$$\rho_{i}\left(r\right)=p_{i}\;\exp(-\alpha_{i}{\left(r-R_{i}\right)}^{2})$$

where:

*ρ*_*i*_(***r***): Shape-density value at a particular location ***r*** for atom *i*.

*p*_*i*_: Gaussian weight.

*α*_*i*_: Decay factor.

***r***: Local coordinate.

***R***_*i*_: Atomic coordinate of atom *i*.

#### 2.6.2 Grouped TWNs-fragment average distance calculation

We used a step-by-step process to calculate the distance between the grouped TWNs and the fragments. Initially, we determined the center coordinate of the fragments. Then, we proceeded to create planes for the grouped TWNs. The process of creating TWN planes for distance calculation employed the four O atom cluster centroids that resulted from the DBSCAN algorithm, which was used for primary grouping of TWNs. By defining the cluster centers as polygon points, we established a plane for each TWN. Consequently, multiple planes were formed within the grouped TWNs. The next step involved calculating the orthogonal distance between each plane of the grouped TWNs and the center coordinate of the fragment. The number of TWNs formed within the various final grouped TWNs may differ. Therefore, to obtain a representative distance, we averaged the orthogonal distances between the planes of the each grouped TWNs and the center coordinate of the fragment. This averaging process ensured a fair representation of the distance between the grouped TWNs and the fragment, taking into account any discrepancies arising from the number of TWNs within the grouped TWNs.

### 2.7 Molecular docking

The X-ray crystal structure of CDK2 (PDB 4ERW) was retrieved and processed as described in the protein preparation section. Molecular docking was carried out on the processed structure using the LigandFit module [72] of Discovery Studio 2022 (BIOVIA, San Diego, CA, USA). The chemical structures of compounds with reported CDK2 inhibitory activity were obtained from the ChEMBL database [73]. The compound structures were energy-minimized using the CHARMm force field. The binding site of the protein was defined based on the co-crystallized ligand, staurosporine. Many kinase inhibitors target the hinge region, which is a crucial recognition site that forms hydrogen bonds with the adenine moiety of ATP. Accordingly, we specified hinge region as the interaction site for hydrogen bonding interactions. A total of one hundred docked poses were generated for each compound and evaluated using the scoring functions. Among these poses, the docking pose with the highest score was selected as the probable binding mode for each compound. For further investigation, the hinge-interacting part of each compound in the predicted binding mode was fragmented in a manner similar to how ligands are fragmented in the KinFragLib [36].

### 2.8 Installation and dependencies

The TWN-FS method requires Python 3.9 or newer installed. We shared the source code on GitHub (https://github.com/pkj0421/TWN-FS) and integrated it into Conda environment. In addition, we provide a yaml file (TWN-env.) that contains all requirements for convenient use by researchers. The provided source codes are that generate the extracted 4-membered ring TWNs into the final grouped TWNs (TWN_gridbox.), and calculates and summarizes the shape similarity and average distance of grouped TWNs-fragments (TWN_analysis.). Furthermore, we also provide source code to automate the process of forming grouped TWNs and calculating the shape similarity and the average distance of grouped TWNs-fragments (TWN_automation.). All source codes can be freely available. The input files are AP site fragments file obtained from KinFragLib and 4-membered ring TWN files extracted from CDK2, which are example files and can be changed based on user analysis. The output files include the file presenting grouped TWNs with only oxygen atoms and a summary file of the shape similarity and average distance results of grouped TWNs-fragments. Additionally, we also provide information about grouped TWNs, the centroid of grouped TWNs, computation log files, and the shape similarity results and average distance results of all grouped TWNs-fragments as output files. The TWN-FS method has a runtime of about 3 min on a single CPU core, from grouped TWNs formation to calculation and analysis.

## 3 Results and discussion

### 3.1 Shape similarity and average distance criteria

KinFragLib is a database specifically developed to aid in FBDD efforts. It serves as a comprehensive fragment library that utilizes the coordinate information from KLIFS database to fragment X-ray crystal ligands. It categorizes fragments based on their specific locations within the kinase binding site including adenine pocket (AP), front pocket (FP), gate area (GA), solvent-exposed pocket (SE), back pocket 1 (B1) and back pocket 2 (B2) site fragments [36]. The library covers protein information for 179 kinases and contains 7486 fragments distributed across different sub-pockets. Among these, 2561 fragments occupy the AP sub-pocket (hinge region). After removing duplicate fragments, it was found 1315 distinct fragments occupy the AP sub-pocket.

We selected 27 human kinases for the examination of protein hot spot locations (Table S1). Among the selected kinases, 25 kinases exhibited a high count (>20) of AP fragments namely CDK2 (330), CHK1 (121), p38a (101), JAK2 (60), ERK2 (77), CK2A1 (58), SYK (59), EGFR (66), GSK3B (45), AURKA (66), BTK (52), BRAF (43), DYRK1A (31), FGFR1 (37), JNK3 (40), LCK (25), CHK2 (29), JAK1 (31), PDK1 (30), TGFBR1 (24), MELK (21), JAK3 (22), WEE1 (20), EPHA2 (26) and HCK (23). Additionally, one kinase showed a moderate count (10−19) of AP fragments namely IGF1R (13) while another kinase displayed a low count (<10) of AP fragments namely ERBB4 (1). We examined the crystal structures of these 27 kinases in the same conformation (DFG-in) where these fragments were observed. For each protein, we analyzed the 4-membered ring TWNs within its binding site and grouped the densely populated TWN locations. The total number of fragments and TWNs observed in AP site of each protein are given in Table S1. We calculated shape similarity and average distance values between grouped TWNs and all the 1315 distinct crystallographic AP sub-pocket fragments to establish the screening criteria. We experimented with various thresholds, including the shape similarity values of ≥ 0.5, ≥ 0.6, and ≥ 0.7, and average distance values of ≤ 0.3, ≤ 0.5, and ≤ 1.0 (Table S2). Subsequently, we determined the filtration rate which represents the count of screened crystallographic AP sub-pocket fragments.

When considering shape similarity value of ≥ 0.5, the percentage range of screened fragments varied based on the average distance value. For an average distance value of ≤ 0.3, the minimum and maximum percentages of screened fragments were 17.6% (IFGF1R) and 58.9% (FGFR1), respectively. With an average distance value of ≤ 0.5, the minimum and maximum percentages were 25.4% (IGF1R) and 73.4% (FGFR1), respectively. Finally, for an average distance value of ≤ 1.0, the minimum and maximum percentages were 37.9% (IGF1R) and 85.5% (LCK), respectively. In summary, when using a shape similarity value of ≥ 0.5, maximum percentage of screened fragments ranged from 58.9% to 85.5% depending on the average distance value. This suggested that a shape similarity value of ≥ 0.5 may not be suitable as a screening threshold due to the high filtration rate of fragments.

When considering shape similarity value of ≥ 0.7, the minimum and maximum percentages of screened fragments for average distance values of ≤ 0.3 were 0.2% (CDK2) and 14.8% (EPHA2), respectively. For average distance value of ≤ 0.5, the minimum and maximum percentages of screened fragments were 1.8% (IGF1R) and 19.9% (ERBB4), respectively. Finally, for average distance value of ≤ 1.0, the minimum and maximum percentages of screened fragments were 2.0% (SYK, IGF1R) and 24.9% (GSK3B), respectively. In summary, when using a shape similarity value of ≥ 0.7, minimum percentage of screened fragments ranged from 0.2% to 2.0% depending on the average distance value. This suggested that a shape similarity value of ≥ 0.7 may not be appropriate as a screening threshold due to the low filtration rate of fragments.

When considering shape similarity values of ≥ 0.6 and an average distance value of ≤ 0.3, we observed that the minimum and maximum percentages of screened fragments were 7.4% (IGF1R) and 39.5% (EPHA2), respectively. Similarly, for shape similarity values of ≥ 0.6 and an average distance value of ≤ 0.5, the minimum and maximum percentages of screened fragments were 10.2% (IGF1R) and 50.6% (EPHA2), respectively. Furthermore, when examining shape similarity values of ≥ 0.6 and an average distance value of ≤ 1.0, the minimum and maximum percentages of screened fragments were 11.6% (IGF1R) and 58.7% (CHK1), respectively. Overall, when considering shape similarity values of ≥ 0.6, the minimum percentage of screened fragments varied from 7.4% to 11.6% while maximum percentage of screened fragments ranged from 39.5% to 58.7% depending on the average distance. Consequently, a shape similarity value of ≥ 0.6 was selected as a screening threshold due to optimum filtration rate of fragments.

Afterwards, we proceeded to determine optimum threshold value for the average distance. We considered unique fragments (fragments occupying the AP sub-pocket of a particular protein) in this process. We analyzed the number of kinases with more than 50.0% of fragments filtered out from each protein, considering a shape similarity of ≥ 0.6. Table 1 shows the number and the percentage of the AP site unique fragments screened from each kinase based on the shape similarity value of ≥ 0.6 and average distance values of ≤ 0.3, ≤ 0.5 and ≤ 1.0. Out of the 27 kinases, 12, 20 and 23 proteins met the criteria when the distance average was ≤ 0.3, ≤ 0.5 and ≤ 1.0, respectively. Consequently, we selected the average distance threshold to be ≤ 0.5 due to moderate filtration rate of fragments. Thus, the screening criteria were established as a shape similarity value of ≥ 0.6 and an average distance value of ≤ 0.5.

**Table 1:** The number and percentage of screened fragments based on shape similarity (S) value of ≥ 0.6 and average distance (D) values of ≤ 0.3, ≤ 0.5 and ≤ 1.0.

Kinase	Unique fragments	S ≥ 0.6		
		D ≤ 0.3	D ≤ 0.5	D ≤ 1.0
CDK2	168	21 (12.5%)	32 (19.0%)	49 (29.2%)
CHK1	87	32 (36.8%)	54 (62.1%)	67 (77.0%)
p38a	70	14 (20.0%)	19 (27.1%)	25 (35.7%)
JAK2	40	28 (70.0%)	34 (85.0%)	38 (95.0%)
ERK2	40	22 (55.0%)	25 (62.5%)	26 (65.0%)
CK2A1	40	4 (10.0%)	6 (15.0%)	18 (45.0%)
SYK	39	25 (64.1%)	28 (71.8%)	29 (74.4%)
EGFR	37	14 (37.8%)	21 (56.8%)	23 (62.2%)
GSK3B	36	10 (27.8%)	16 (44.4%)	23 (63.9%)
AURKA	35	17 (48.6%)	22 (62.9%)	24 (68.6%)
BTK	33	19 (57.6%)	23 (69.7%)	26 (78.8%)
BRAF	28	13 (46.4%)	20 (71.4%)	22 (78.6%)
DYRK1A	28	18 (64.3%)	20 (71.4%)	23 (82.1%)
FGFR1	28	12 (42.9%)	18 (64.3%)	24 (85.7%)
JNK3	27	13 (48.1%)	14 (51.9%)	14 (51.9%)
LCK	24	2 (8.3%)	5 (20.8%)	14 (58.3%)
CHK2	23	13 (56.5%)	14 (60.9%)	14 (60.9%)
JAK1	21	18 (85.7%)	18 (85.7%)	18 (85.7%)
PDK1	20	11 (55.0%)	13 (65.0%)	13 (65.0%)
TGFBR1	19	15 (78.9%)	15 (78.9%)	16 (84.2%)
MELK	17	1 (5.9%)	1 (5.9%)	2 (11.8%)
JAK3	12	3 (25.0%)	10 (83.3%)	11 (91.7%)
WEE1	11	2 (18.2%)	4 (36.4%)	7 (63.6%)
IGF1R	9	5 (55.6%)	8 (88.9%)	8 (88.9%)
EPHA2	8	4 (50.0%)	6 (75.0%)	7 (87.5%)
HCK	5	3 (60.0%)	3 (60.0%)	4 (80.0%)
ERBB4	1	0 (0.0%)	1 (100.0%)	1 (100.0%)

### 3.2 Location of grouped TWNs

**Fig. 4:**
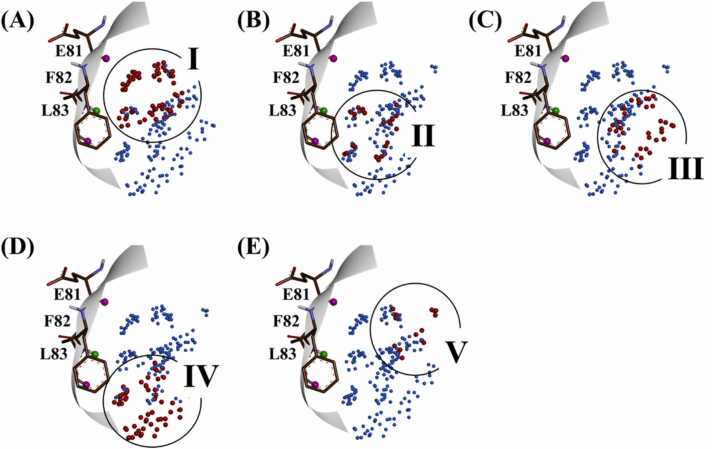
Grouped TWNs in five specific regions within the AP site of CDK2. (A) Region I, (B) region II, (C) region III, (D) region IV and (E) region V. Hinge residues E81, F82 and L83 are shown as thin stick models. The hydrogen bond acceptor of the hinge residue amide backbone is represented by the pink ball while the green ball represents the hydrogen bond donor.

In the KinFragLib database, CDK2 showed highest count of the AP site unique fragments (168) among all kinases (Table 1). Accordingly, we selected CDK2 as the target protein for further analysis. As shown in Fig. 4, grouped TWNs were observed in five specific regions (I-V) within the AP site of CDK2. Detailed analysis of various regions revealed that 127 fragments out of 168 AP site unique fragments were located in the region I, while regions II, III, IV and V contained 13, 7, 14 and 7 fragments respectively. Furthermore, out of the 168 AP site unique fragments, 32 fragments were screened through the TWN-FS method. Among the 32 fragments that were screened, 29, 1 and 2 fragments were located in the regions I, II and V respectively. These findings suggested that region I might be the hot spot within the AP site of CDK2.

### 3.3 Screening of CDK2 inhibitors

**Fig. 5:**
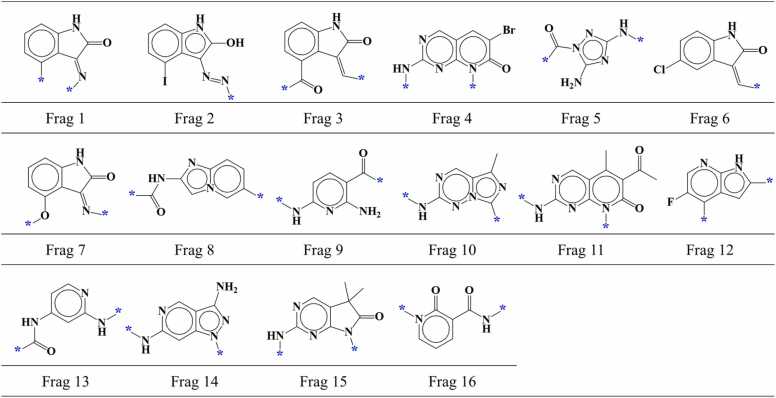
AP site fragments from active compounds with an IC_50_ value of ≤ 1000 nM for CDK2. The * symbol indicates the linking part.

**Fig. 6:**
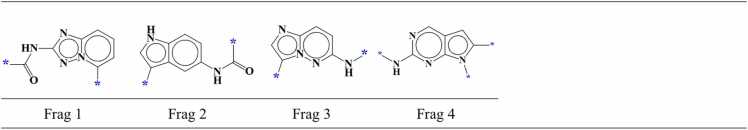
AP site fragments from inactive compounds with an IC_50_ value of ≥ 10,000 nM for CDK2. The * symbol indicates the linking part.

We screened known CDK2 inhibitors using TWN-FS method considering the region I within the AP site of CDK2 to investigate its applicability as a FBDD screening method. A total of 2026 compounds with IC_50_ values for the CDK2 single protein were obtained from the ChEMBL database. To focus on small molecules that could be fragmented in a manner similar to the ligands in the KinFragLib, we excluded compounds with a molecular weight exceeding 500, as well as compounds like staurosporine and macromolecules from the obtained compounds. Furthermore, to ensure that screening results include fragments other than crystallographic CDK2 fragments, compounds containing fragments identical to those found in the KinFragLib were excluded. Finally, a total of 1016 compounds were selected including 571 active compounds with an IC_50_ value of ≤ 1000 nM and 445 inactive compounds with an IC_50_ value of ≥ 10,000 nM. Using molecular docking, we predicted the binding modes of these compounds to the CDK2 and conducted an analysis of the predicted binding modes with a particular focus on the hinge region (AP site). The hinge-interacting part of each compound in the predicted binding mode was fragmented in a manner similar to the fragmentation process used for ligands in the KinFragLib. Out of 1016 fragments, 228 fragments were found to be located in the region I within the AP site of CDK2. Shape similarity and average distance values were calculated between these fragments and grouped TWNs observed in the region I. Using the TWN-FS method, 37 fragments were screened from a total of 228 fragments with the established screening criteria. These fragments included 19 fragments from active compounds and 18 fragments from inactive compounds. Detailed information about these screened fragments including fragment structure, ChEMBL ID of compounds, IC_50_ value, predicted binding mode, docking score, shape similarity value and average distance value are presented in Tables S3 and S4. After categorizing identical fragments, we found that 37 screened fragments could be classified into 20 different types including 16 fragment types from active compounds (Fig. 5, Fig. 4 fragment types from inactive compounds (Fig. 6).

### 3.4 Screening of CHK1, IGF1R and ERBB4 inhibitors

**Fig. 7:**
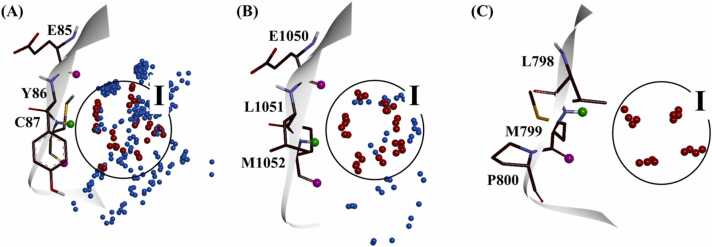
Grouped TWNs in the region I within the AP site of various kinases. (A) CHK1, (B) IGF1R and (C) ERBB4. Hinge residues of each kinase are shown as thin stick models. The hydrogen bond acceptor of the hinge residue amide backbone is represented by the pink ball while the green ball represents the hydrogen bond donor.

In order to assess the potential of the TWN-FS method more comprehensively, we screened known kinase inhibitors targeting a broader range of kinases. In the KinFragLib database, CHK1, IGF1R and ERBB4 showed high (87), moderate (9) and low (1) count of the AP site unique fragments, respectively (Table 1). Accordingly, we selected these kinases and analyzed grouped TWNs in the region I within their AP sites (Fig. 7). The known inhibitors of CHK1, IGF1R and ERBB4 were obtained from the ChEMBL database, classified as active and inactive compounds and screened using TWN-FS method in a similar way as described for CDK2.

**Fig. 8:**
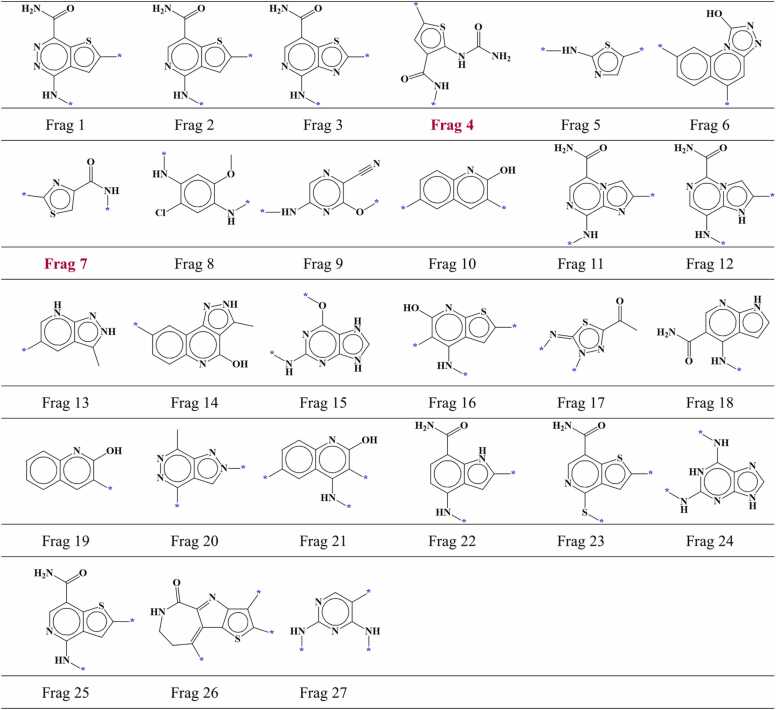
AP site fragments from active compounds with an IC_50_ value of ≤ 1000 nM for CHK1. The * symbol indicates the linking part. Fragments common between the active and inactive compounds (Fig. 9) are highlighted in dark red.

**Fig. 9:**
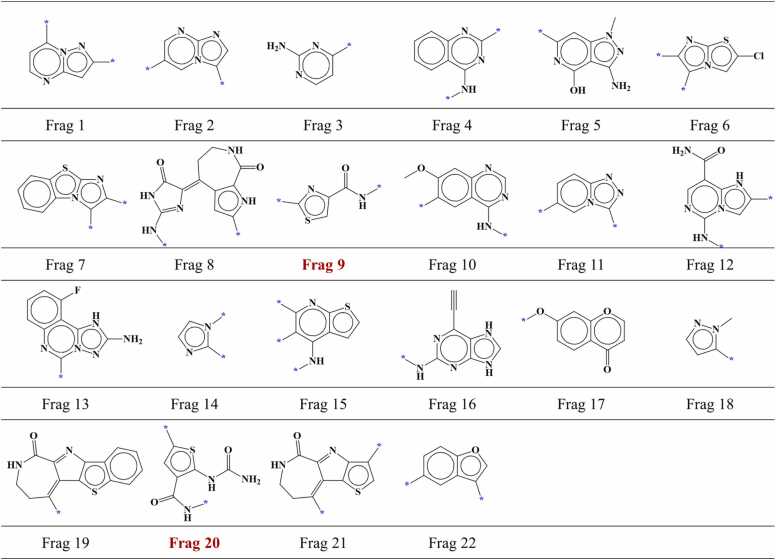
AP site fragments from inactive compounds with an IC_50_ value of ≥ 10,000 nM for CHK1. The * symbol indicates the linking part. Fragments common between the inactive and active compounds (Fig. 8) are highlighted in dark red.

For CHK1, 986 compounds were chosen which included 743 active compounds and 243 inactive compounds. These compounds were docked inside the binding site of CHK1 and hinge-interacting part of each compound was fragmented. Among 986 fragments, 356 were located in the region I within the AP site of CHK1. Out of 356 fragments, 175 fragments were screened with the established screening criteria which included 125 fragments from active compounds and 50 fragments from inactive compounds. Subsequent analysis of identical fragments revealed that the 175 screened compounds could be classified into 49 different fragment types comprising 27 fragment types from active compounds (Fig. 8) and 22 fragment types from inactive compounds (Fig. 9). Furthermore, 2 fragment types were identified as common between the active and inactive compound fragment types.

**Fig. 10:**
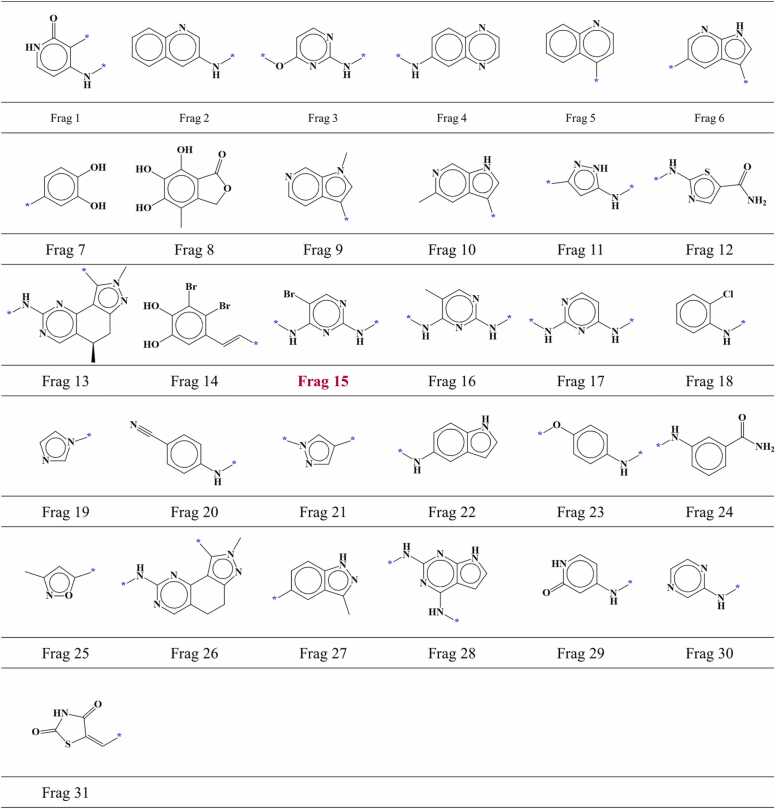
AP site fragments from active compounds with an IC_50_ value of ≤ 1000 nM for IGF1R. The * symbol indicates the linking part. Fragment common between the active and inactive compounds (Fig. 11) are highlighted in dark red.

**Fig. 11:**
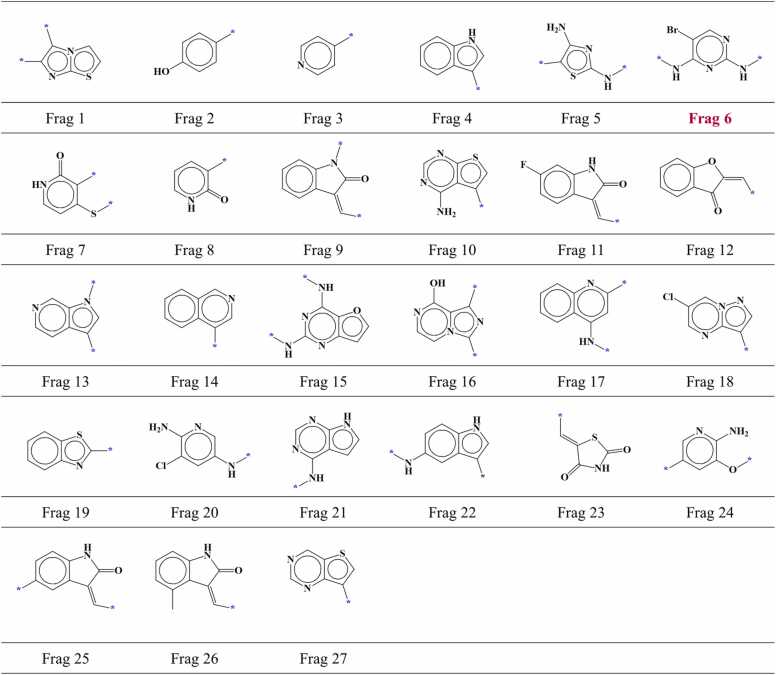
AP site fragments from inactive compounds with an IC_50_ value of ≥ 10,000 nM for IGF1R. The * symbol indicates the linking part. Fragments common between the inactive and active compounds (Fig. 10) are highlighted in dark red.

A total of 1126 compounds (795 active and 331 inactive compounds) were selected for IGF1R. These compounds were subjected to docking within the IGF1R's binding site, and the hinge-interacting portion of each compound was subsequently fragmented. Out of 1126 fragments, 280 fragments were specifically situated in region I within the AP site of IGF1R. From these 280 fragments, 175 fragments were screened based on the established screening criteria. Screened fragments comprised of 118 fragments from active compounds and 57 fragments from inactive compounds. Upon thorough examination of identical fragments, we identified 58 different fragment types among these 175 screened fragments including 31 fragment types from the active compounds (Fig. 10) and 27 fragment types from the inactive compounds (Fig. 11). Additionally, 1 fragment type was found to be common between the active and inactive compound fragment types.

**Fig. 12:**
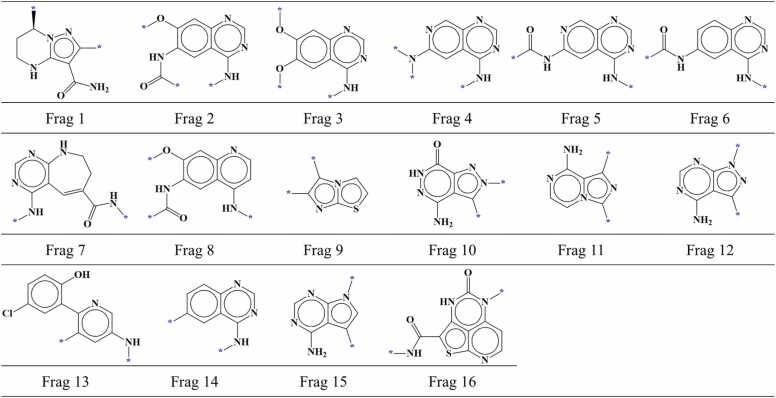
AP site fragments from active compounds with an IC_50_ value of ≤ 1000 nM for ERBB4. The * symbol indicates the linking part.

**Fig. 13:**
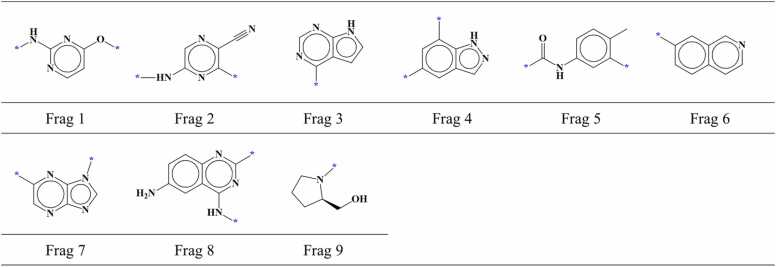
AP site fragments from inactive compounds with an IC_50_ value of ≥ 10,000 nM for ERBB4. The * symbol indicates the linking part.

For ERBB4, total 157 compounds comprising of 119 active compounds and 38 inactive compounds were selected. Compounds were docked in the binding site of ERBB4 and the part of each compound that interacted with the hinge region was fragmented. Among 157 fragments, 81 were positioned in the region I within the AP site of ERBB4. Using the established screening criteria, 77 fragments were screened out of these 81 fragments. Screened fragments contained 67 fragments from active compounds and 10 fragments from inactive compounds. When we examined the identical fragments, we found that 77 screened compounds could be categorized into 25 different fragment types including 16 from active compounds (Fig. 12, Fig. 9 from inactive ones (Fig. 13).

In summary, fragments from active (IC_50_ value of ≤1000 nM) and inactive compounds (IC_50_ value of ≥10,000 nM) for various kinases were screened using the TWN-FS method. In the screening results, fragments from active and inactive compounds were 19 and 18 for CDK2, 125 and 50 for CHK1, 118 and 57 for IGF1R, and 67 and 10 for ERBB4, respectively. Overall, fragments from active compounds were found to be more than fragments from inactive compounds. Subsequent analysis of identical fragments showed that fragment types from active and inactive compounds were 16 and 4 for CDK2, 27 and 22 for CHK1, and 31 and 27 for IGF1R, and 16 and 9 for ERBB4, respectively. This revealed that number of fragment types from active compounds was comparatively higher. These results suggest that TWN-FS method has the potential to effectively screen diverse fragments based on a specific region within the protein binding site. Later, screened fragment hits could be optimized into potent leads by considering other regions of the binding site.

## 4 Conclusions

We present TWN-FS method, a novel method for fragment screening. This method analyzes water networks namely TWNs in the protein binding site without relying on computationally expensive explicit free energy calculations and group spatially close TWNs. The locations of grouped TWNs may indicate hot spots in the binding site. TWN-FS method considers shape and geometry of the grouped TWNs. The core principle of screening involves calculating and comparing the shape similarity and average distance values between grouped TWNs and fragments. The threshold values for shape similarity and average distance used in this method are based on the TWNs observed in the binding sites of several kinases and all known crystallographic kinase fragments. One drawback of TWN-FS method is that the results can be significantly influenced by the binding pose of the fragments. We screened known CDK2, CHK1, IGF1R and ERBB4 inhibitors using the TWN-FS method specifically targeting a specific location of the hinge region (AP site) to explore the effectiveness of this approach in fragment screening. The outcomes of this study suggest that TWN-FS method holds significant potential in efficiently screening fragments for FBDD. Furthermore, this method has the capability to suggest fragment hits by focusing on a specific region within the binding site instead of the entire binding site. These hits can then be effectively optimized into potent leads by taking into account other regions of the binding site.

## Declaration of Competing Interest

The authors declare that they have no known competing financial interests or personal relationships that could have appeared to influence the work reported in this paper.

## Data Availability

The codes used for this research work are made publicly available in the GitHub repository [https://github.com/pkj0421/TWN-FS].
