# Supplementary material for: TWN-FS method: A novel fragment screening method for drug discovery
Source: Comput Struct Biotechnol J. 2023 Sep 29;21:4683–96. doi: 10.1016/j.csbj.2023.09.037 (PMC10568351; doi:10.1016/j.csbj.2023.09.037)
Supplement: Supplementary file 1 — Supplementary material [file mmc1.docx]

**Table S1**

Summary of fragments and TWNs observed in the AP site of various proteins.

| Kinase | Protein | PDB code | Resolution (Å) | Units^#^ | TWNs^#^ | Grouped TWNs^#^ | AP site fragments^#^ | Unique fragments^#^ |
| --- | --- | --- | --- | --- | --- | --- | --- | --- |
| CDK2 | Cyclin Dependent Kinase 2 | 4ERW | 2.00 | 22,244 | 9,039 | 898 | 330 | 168 |
| CHK1 | Checkpoint Kinase 1 | 1NVR | 1.80 | 32,281 | 12,760 | 726 | 121 | 87 |
| p38a | Mitogen-Activated Protein Kinase 14 | 4F9Y | 1.85 | 22,330 | 8,886 | 643 | 101 | 70 |
| JAK2 | Janus Kinase 2 | 4IVA | 1.50 | 23,615 | 9,428 | 594 | 60 | 40 |
| ERK2 | Mitogen-Activated Protein Kinase 1 | 4ZZN | 1.33 | 23,857 | 9,855 | 1,467 | 77 | 40 |
| CK2A1 | Casein Kinase 2 Alpha 1 | 5T1H | 2.11 | 26,995 | 10,916 | 1,586 | 58 | 40 |
| SYK | Spleen Associated Tyrosine Kinase | 1XBC | 2.00 | 27,181 | 10,503 | 520 | 59 | 39 |
| EGFR | Epidermal Growth Factor Receptor | 4ZAU | 2.80 | 26,771 | 10,656 | 653 | 66 | 37 |
| GSK3B | Receptor (EGFR), Glycogen Synthase Kinase 3 Beta | 1Q3D | 2.20 | 31,051 | 12,390 | 696 | 45 | 36 |
| AURKA | Aurora Kinase A | 5DT0 | 2.15 | 23,492 | 9,230 | 468 | 66 | 35 |
| BTK | Bruton Tyrosine Kinase | 6X3N | 1.95 | 27,255 | 10,669 | 529 | 52 | 33 |
| BRAF | B-Raf Proto-Oncogene, Serine/Threonine Kinase | 3Q4C | 3.20 | 24,598 | 9,982 | 469 | 43 | 28 |
| DYRK1A | Dual Specificity Tyrosine Phosphorylation Regulated Kinase 1A | 4NCT | 2.60 | 29,811 | 12,179 | 915 | 31 | 28 |
| FGFR1 | Fibroblast Growth Factor Receptor 1 | 5EW8 | 1.63 | 26,550 | 10,748 | 674 | 37 | 28 |
| JNK3 | Mitogen-Activated Protein Kinase 10 | 4Z9L | 2.10 | 20,168 | 8,233 | 1,557 | 40 | 27 |
| LCK | LCK Proto-Oncogene, Src Family Tyrosine Kinase | 3AC1 | 1.99 | 30,975 | 12,774 | 946 | 25 | 24 |
| CHK2 | Checkpoint Kinase 2 | 2CN5 | 2.25 | 34,991 | 13,740 | 1,165 | 29 | 23 |
| JAK1 | Janus Kinase 1 | 6N7A | 1.33 | 23,550 | 9,550 | 642 | 31 | 21 |
| PDK1 | Pyruvate Dehydrogenase Kinase 1 | 1OKY | 2.30 | 29,083 | 11,331 | 417 | 30 | 20 |
| TGFBR1 | Transforming Growth Factor Beta Receptor 1 | 5QIM | 1.75 | 25,084 | 9,964 | 717 | 24 | 19 |
| MELK | Maternal Embryonic Leucine Zipper Kinase | 5M5A | 1.90 | 22,298 | 8,870 | 815 | 21 | 17 |
| JAK3 | Janus Kinase 3 | 5LWM | 1.55 | 25,273 | 10,327 | 1,048 | 22 | 12 |
| WEE1 | WEE1 G2 Checkpoint Kinase | 1X8B | 1.81 | 27,984 | 11,341 | 599 | 20 | 11 |
| IGF1R | Insulin Like Growth Factor 1 Receptor | 3NW6 | 2.20 | 29,039 | 11,937 | 1,615 | 13 | 9 |
| EPHA2 | EPH Receptor A2 | 5IA2 | 1.62 | 26,221 | 10,525 | 598 | 26 | 8 |
| HCK | HCK Proto-Oncogene, Src Family Tyrosine Kinase | 2HK5 | 2.00 | 32,365 | 13,349 | 692 | 23 | 5 |
| ERBB4 | Erb-B2 Receptor Tyrosine Kinase 4 | 2R4B | 2.40 | 24,237 | 9,769 | 841 | 1 | 1 |

Columns marked with # represent counts. Unique fragments refer to fragments occupying the AP sub-pocket of a particular protein after removing duplicates.

**Table S2**

The number and percentage of screened fragments based on different shape similarity (S) and average distance (D) values between all the 1,315 distinct crystallographic AP sub-pocket fragments and the grouped TWNs within AP site of each protein.

| Kinase | S ≥0.5 | | | S ≥0.6 | | | S ≥0.7 | | |
| --- | --- | --- | --- | --- | --- | --- | --- | --- | --- |
|  | D ≤0.3 | D ≤0.5 | D ≤1.0 | D ≤0.3 | D ≤0.5 | D ≤1.0 | D ≤0.3 | D ≤0.5 | D ≤1.0 |
| CDK2 | 280(21.3%) | 504 (38.3%) | 918 (69.8%) | 99 (7.5%) | 217 (16.5%) | 496 (37.7%) | 2 (0.2%) | 25 (1.9%) | 113 (8.6%) |
| CHK1 | 471 (35.8%) | 740 (56.3%) | 1089 (82.8%) | 319 (24.3%) | 523 (39.8%) | 772 (58.7%) | 49 (3.7%) | 115 (8.7%) | 185 (14.1%) |
| p38a | 451 (34.3%) | 635 (48.3%) | 865 (65.8%) | 294 (22.4%) | 409 (31.1%) | 507 (38.6%) | 78 (5.9%) | 97 (7.4%) | 108 (8.2%) |
| JAK2 | 436 (33.2%) | 691 (52.5%) | 1084 (82.4%) | 340 (25.9%) | 529 (40.2%) | 768 (58.4%) | 155 (11.8%) | 235 (17.9%) | 289 (22.0%) |
| ERK2 | 626 (47.6%) | 802 (61.0%) | 916 (69.7%) | 363 (27.6%) | 443 (33.7%) | 478 (36.3%) | 44 (3.3%) | 68 (5.2%) | 77 (5.9%) |
| CK2A1 | 303 (23.0%) | 495 (37.6%) | 923 (70.2%) | 112 (8.5%) | 227 (17.3%) | 502 (38.2%) | 10 (0.8%) | 57 (4.3%) | 140 (10.6%) |
| SYK | 644 (49.0%) | 871 (66.2%) | 1078 (82.0%) | 361 (27.5%) | 477 (36.3%) | 579 (44.0%) | 17 (1.3%) | 25 (1.9%) | 26 (2.0%) |
| EGFR | 504 (38.3%) | 698 (53.1%) | 891 (67.8%) | 229 (17.4%) | 339 (25.8%) | 424 (32.2%) | 50 (3.8%) | 78 (5.9%) | 110 (8.4%) |
| GSK3B | 430 (32.7%) | 653 (49.7%) | 1107 (84.2%) | 261 (19.8%) | 401 (30.5%) | 759 (57.7%) | 61 (4.6%) | 127 (9.7%) | 327 (24.9%) |
| AURKA | 617 (46.9%) | 842 (64.0%) | 1035 (78.7%) | 350 (26.6%) | 475 (36.1%) | 553 (42.1%) | 43 (3.3%) | 67 (5.1%) | 81 (6.2%) |
| BTK | 620 (47.1%) | 811 (61.7%) | 932 (70.9%) | 346 (26.3%) | 418 (31.8%) | 478 (36.3%) | 55 (4.2%) | 76 (5.8%) | 85 (6.5%) |
| BRAF | 664 (50.5%) | 873 (66.4%) | 1042 (79.2%) | 468 (35.6%) | 613 (46.6%) | 697 (53.0%) | 180 (13.7%) | 237 (18.0%) | 271 (20.6%) |
| DYRK1A | 660 (50.2%) | 911 (69.3%) | 1085 (82.5%) | 467 (35.5%) | 612 (46.5%) | 689 (52.4%) | 175 (13.3%) | 213 (16.2%) | 224 (17%) |
| FGFR1 | 775 (58.9%) | 965 (73.4%) | 1105 (84.0%) | 501 (38.1%) | 617 (46.9%) | 691 (52.5%) | 81 (6.2%) | 115 (8.7%) | 135 (10.3%) |
| JNK3 | 366 (27.8%) | 492 (37.4%) | 635 (48.3%) | 156 (11.9%) | 198 (15.1%) | 223 (17.0%) | 29 (2.2%) | 32 (2.4%) | 33 (2.5%) |
| LCK | 636 (48.4%) | 890 (67.7%) | 1124 (85.5%) | 437 (33.2%) | 603 (45.9%) | 764 (58.1%) | 110 (8.4%) | 183 (13.9%) | 245 (18.6%) |
| CHK2 | 642 (48.8%) | 897 (68.2%) | 1087 (82.7%) | 348 (26.5%) | 486 (37.0%) | 570 (43.3%) | 64 (4.9%) | 96 (7.3%) | 107 (8.1%) |
| JAK1 | 606 (46.1%) | 813 (61.8%) | 1046 (79.5%) | 444 (33.8%) | 571 (43.4%) | 689 (52.4%) | 116 (8.8%) | 156 (11.9%) | 174 (13.2%) |
| PDK1 | 716 (54.4%) | 931 (70.8%) | 1057 (80.4%) | 426 (32.4%) | 529 (40.2%) | 589 (44.8%) | 106 (8.1%) | 136 (10.3%) | 151 (11.5%) |
| TGFBR1 | 644 (49.0%) | 882 (67.1%) | 1062 (80.8%) | 389 (29.6%) | 493 (37.5%) | 555 (42.2%) | 79 (6.0%) | 102 (7.8%) | 113 (8.6%) |
| MELK | 473 (36.0%) | 680 (51.7%) | 1008 (76.7%) | 185 (14.1%) | 327 (24.9%) | 553 (42.1%) | 23 (1.7%) | 63 (4.8%) | 153 (11.6%) |
| JAK3 | 264 (20.1%) | 499 (37.9%) | 1022 (77.7%) | 193 (14.7%) | 368 (28.0%) | 695 (52.9%) | 64 (4.9%) | 130 (9.9%) | 226 (17.2%) |
| WEE1 | 316 (24.0%) | 553 (42.1%) | 1039 (79.0%) | 192 (14.6%) | 367 (27.9%) | 681 (51.8%) | 43 (3.3%) | 83 (6.3%) | 115 (8.7%) |
| IGF1R | 231 (17.6%) | 334 (25.4%) | 499 (37.9%) | 97 (7.4%) | 134 (10.2%) | 152 (11.6%) | 18 (1.4%) | 24 (1.8%) | 26 (2.0%) |
| EPHA2 | 721 (54.8%) | 941 (71.6%) | 1108 (84.3%) | 520 (39.5%) | 665 (50.6%) | 754 (57.3%) | 195 (14.8%) | 252 (19.2%) | 287 (21.8%) |
| HCK | 620 (47.1%) | 776 (59.0%) | 897 (68.2%) | 344 (26.2%) | 428 (32.5%) | 504 (38.3%) | 66 (5.0%) | 99 (7.5%) | 132 (10.0%) |
| ERBB4 | 424 (32.2%) | 687 (52.2%) | 1070 (81.4%) | 330 (25.1%) | 529 (40.2%) | 741 (56.3%) | 163 (12.4%) | 262 (19.9%) | 290 (22.1%) |

**Table S3**

Details of 19 AP site fragments from active compounds with an IC_50_ value of ≤1,000 nM screened using the TWN-FS method.

| ChEMBL ID | IC_50_ value (nM) for CDK2 | Binding  mode to CDK2 | Fragment  structure | Docking  score | Shape  similarity | Average distance |
| --- | --- | --- | --- | --- | --- | --- |
| ChEMBL  411224 | 1 | 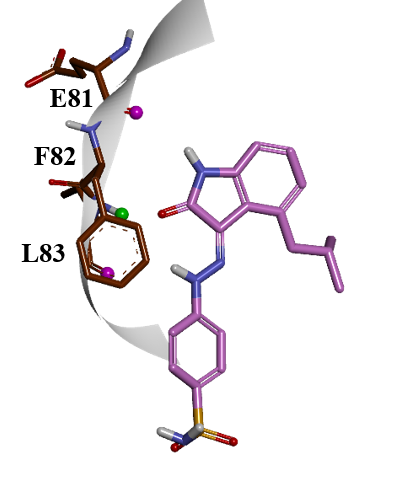 |  | 81.89 | 0.61 | 0.25 |
| ChEMBL  314397 | 5 | 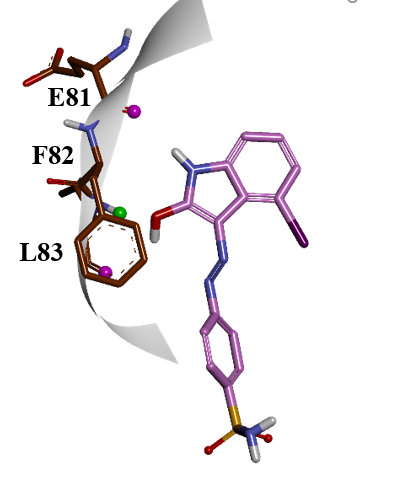 |  | 75.25 | 0.65 | 0.21 |
| ChEMBL  408210 | 9 | 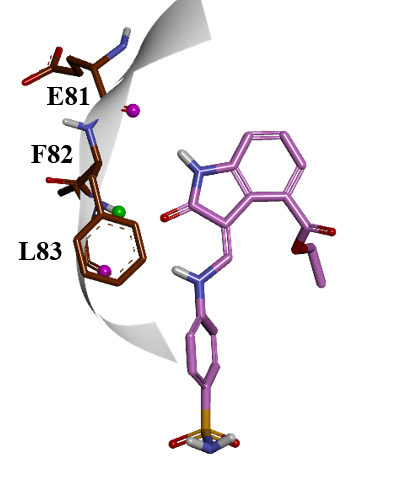 |  | 80.73 | 0.60 | 0.49 |
| ChEMBL  1794054 | 21 | 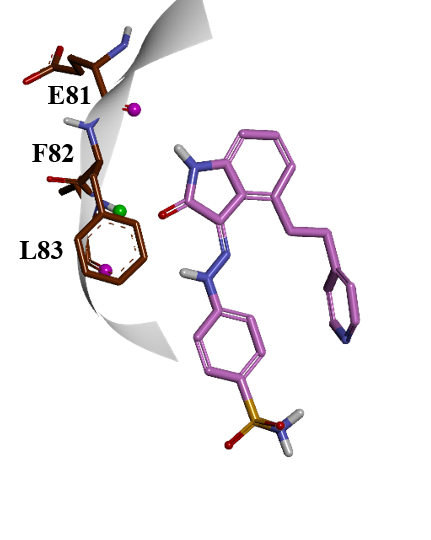 |  | 74.69 | 0.64 | 0.27 |
| ChEMBL  190281 | 43 | 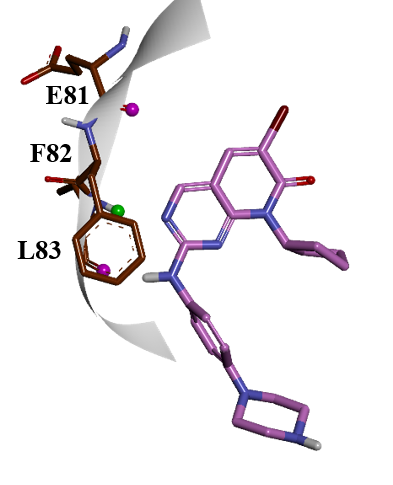 |  | 72.78 | 0.61 | 0.34 |
| ChEMBL  209280 | 71 | 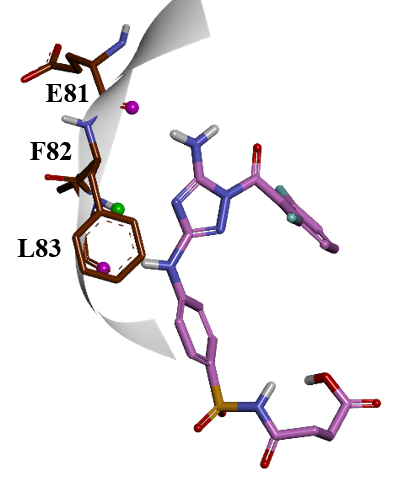 |  | 98.11 | 0.61 | 0.20 |
| ChEMBL  301639 | 88 | 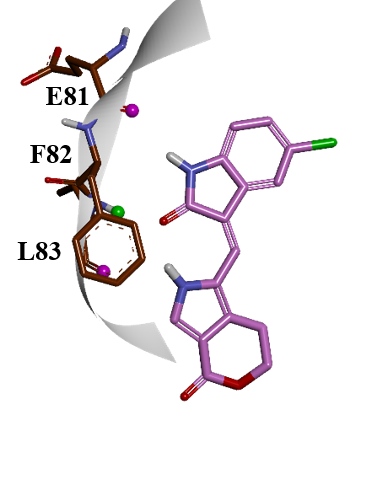 |  | 61.08 | 0.64 | 0.38 |
| ChEMBL  411696 | 93 | 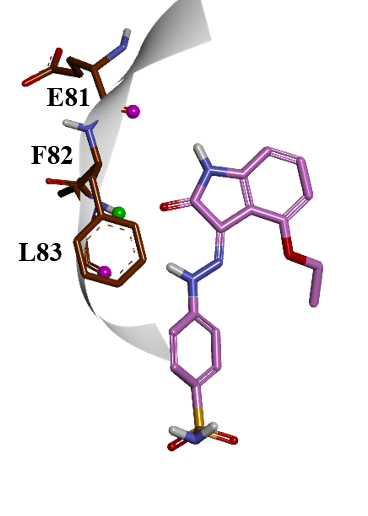 |  | 85.52 | 0.60 | 0.43 |
| ChEMBL  212264 | 140 | 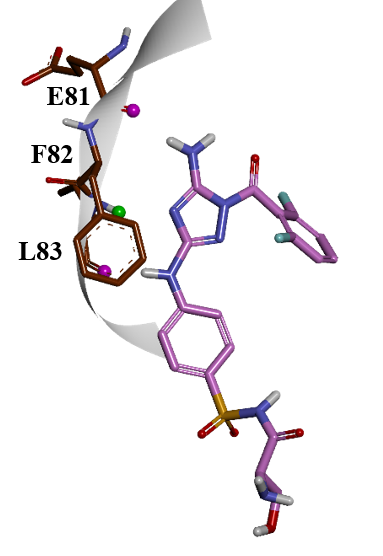 |  | 68.68 | 0.60 | 0.11 |
| ChEMBL  1642655 | 155 | 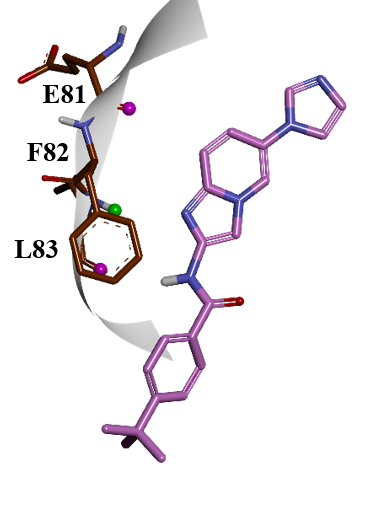 |  | 55.17 | 0.60 | 0.03 |
| ChEMBL  193643 | 180 | 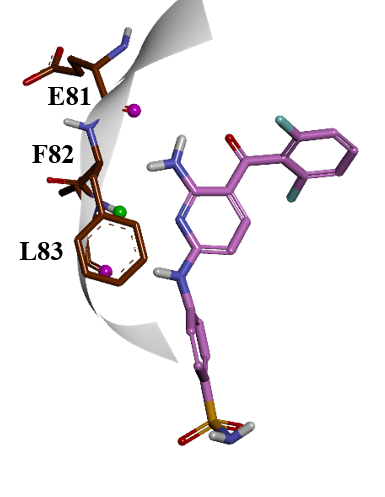 |  | 72.73 | 0.63 | 0.10 |
| ChEMBL  519715 | 230 | 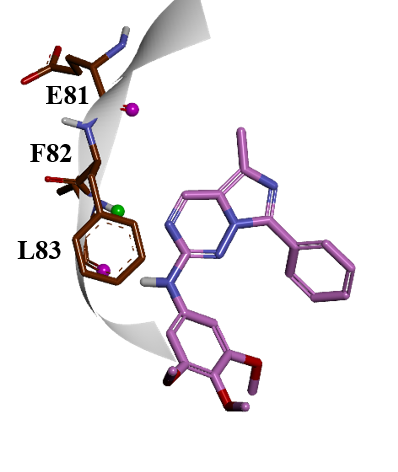 |  | 66.30 | 0.62 | 0.33 |
| ChEMBL  365847 | 230 | 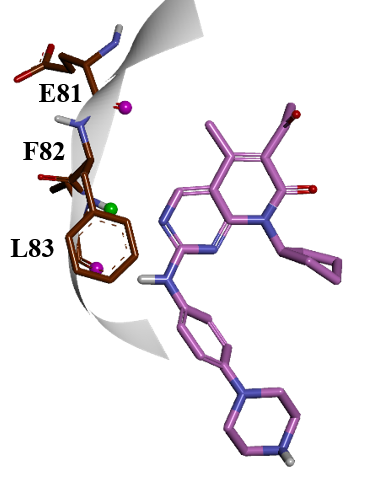 |  | 69.86 | 0.61 | 0.22 |
| ChEMBL  4777981 | 320 | 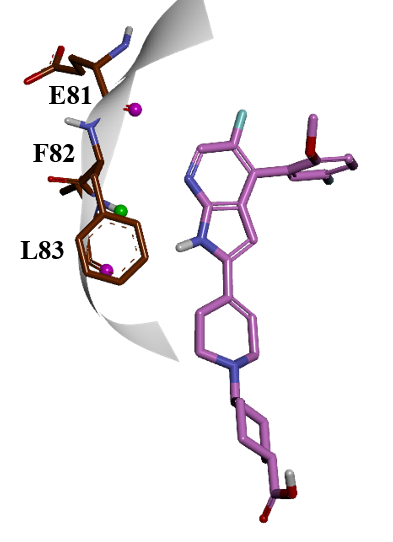 |  | 53.19 | 0.60 | 0.27 |
| ChEMBL  193456 | 500 | 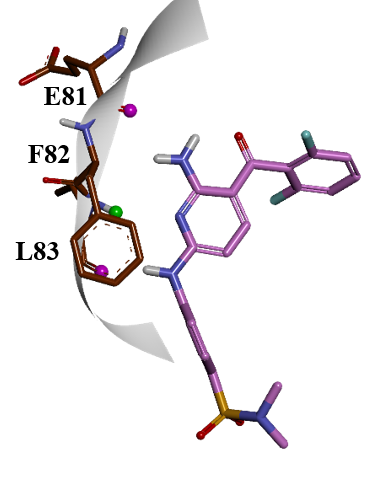 |  | 63.58 | 0.63 | 0.11 |
| ChEMBL  3352835 | 503 | 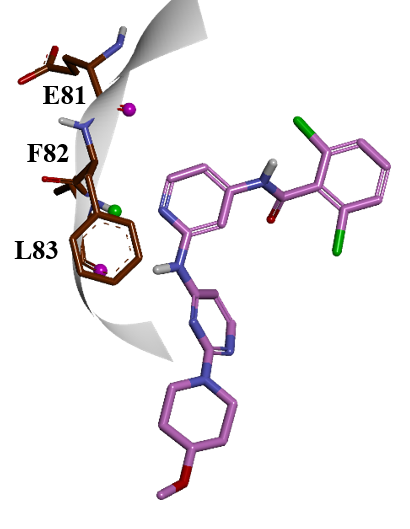 |  | 71.85 | 0.60 | 0.25 |
| ChEMBL  3354191 | 530 | 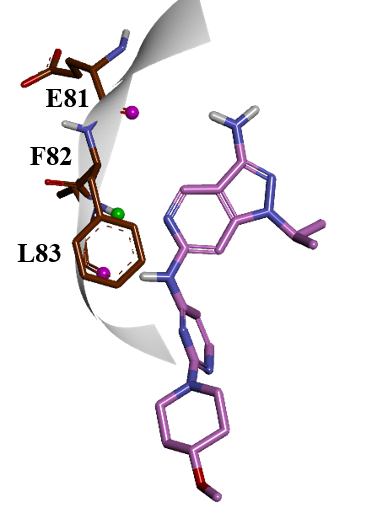 |  | 71.69 | 0.63 | 0.12 |
| ChEMBL  3884319 | 605 | 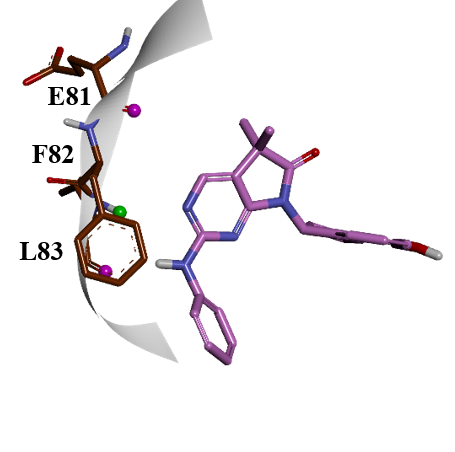 |  | 62.19 | 0.60 | 0.42 |
| ChEMBL  509101 | 1,000 | 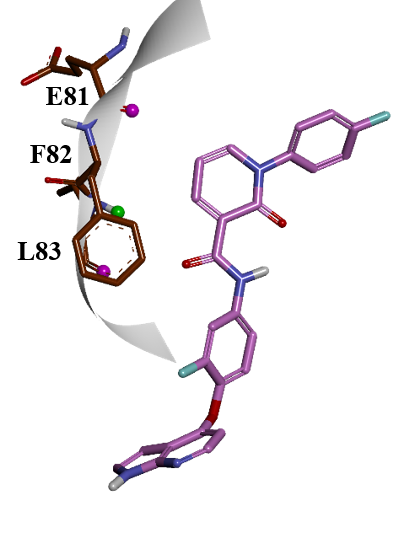 |  | 64.33 | 0.60 | 0.23 |

The * symbol in fragment structure indicates the linking part.

**Table S4**

Details of 18 AP site fragments from inactive compounds with an IC_50_ value of ≥10,000 nM screened using the TWN-FS method.

| ChEMBL ID | IC_50_ value (nM) for CDK2 | Binding  Mode to CDK2 | Fragment structure | Docking  score | Shape  similarity | Average  distance |
| --- | --- | --- | --- | --- | --- | --- |
| ChEMBL  3360318 | 10,000 | 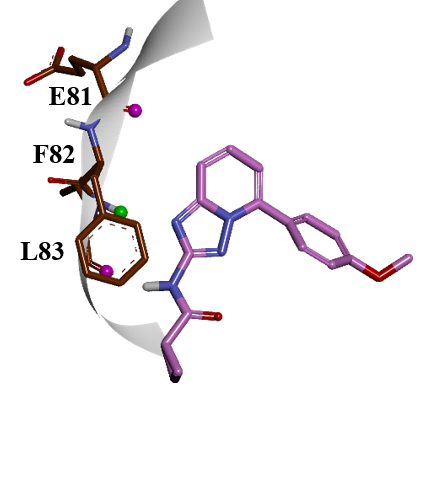 |  | 58.90 | 0.60 | 0.17 |
| ChEMBL  2062581 | 14,000 | 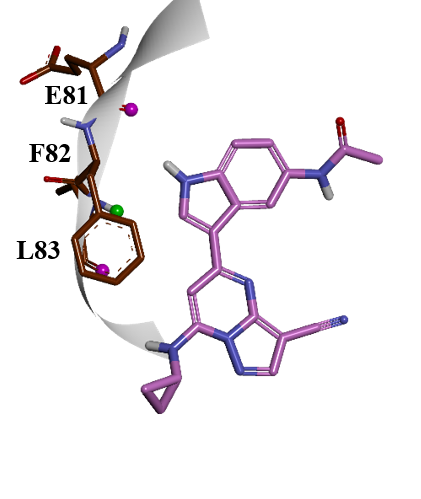 |  | 66.98 | 0.60 | 0.22 |
| ChEMBL  3658050 | 15,000 | 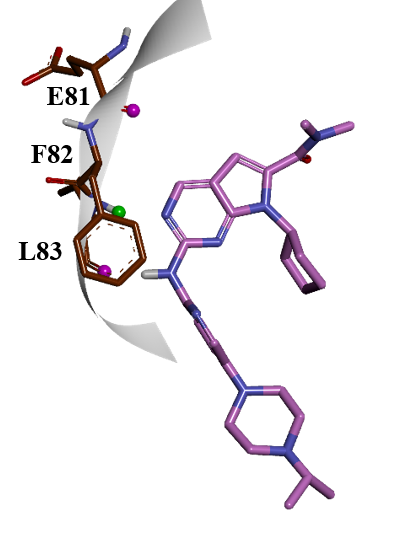 |  | 69.14 | 0.61 | 0.25 |
| ChEMBL  3658049 | 15,000 | 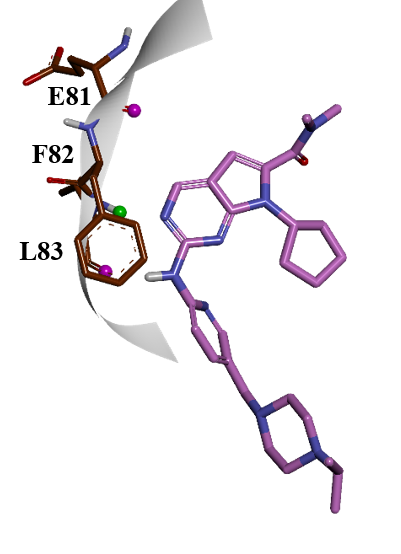 | 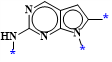 | 69.98 | 0.64 | 0.47 |
| ChEMBL  3658084 | 15,000 | 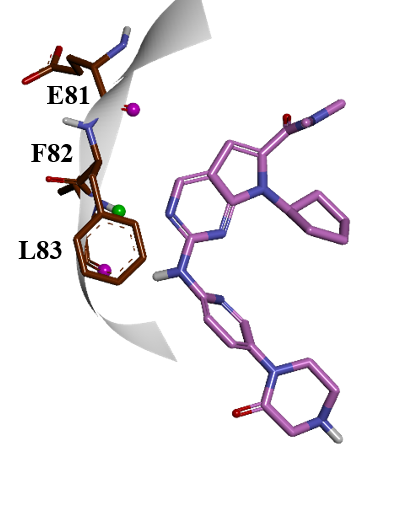 |  | 70.46 | 0.62 | 0.16 |
| ChEMBL  3658024 | 15,000 | 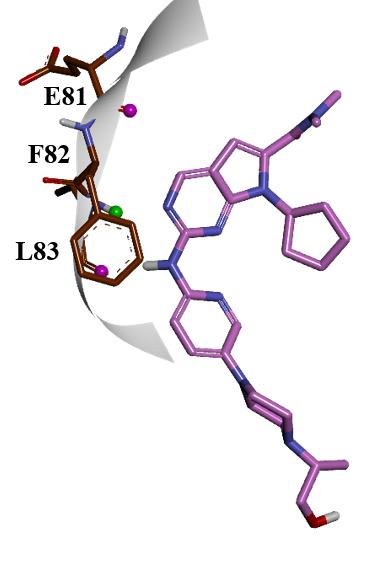 |  | 70.74 | 0.62 | 0.19 |
| ChEMBL  3545110 | 15,000 | 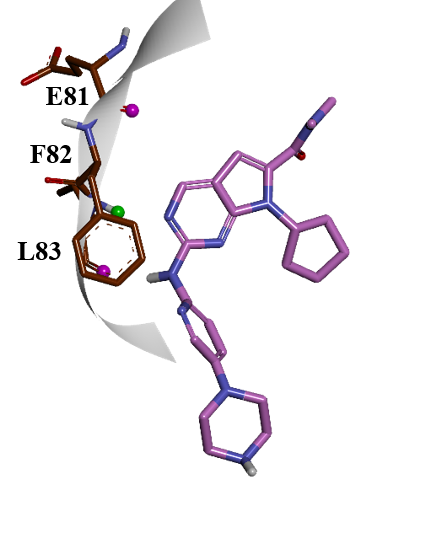 |  | 71.26 | 0.60 | 0.13 |
| ChEMBL  3658053 | 15,000 | 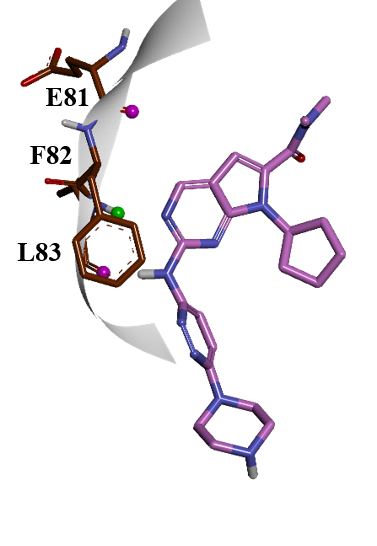 |  | 71.39 | 0.61 | 0.23 |
| ChEMBL  3654246 | 15,000 | 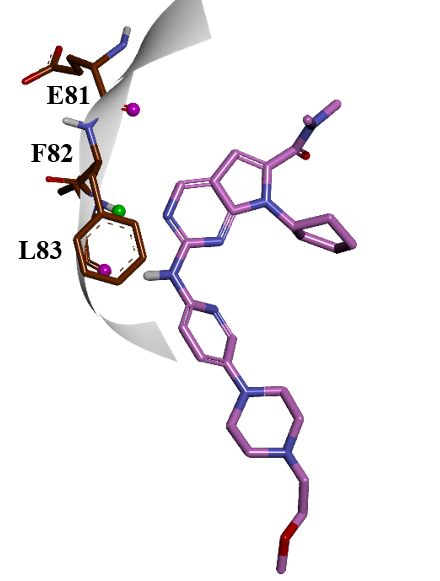 |  | 71.60 | 0.60 | 0.22 |
| ChEMBL  3658043 | 15,000 | 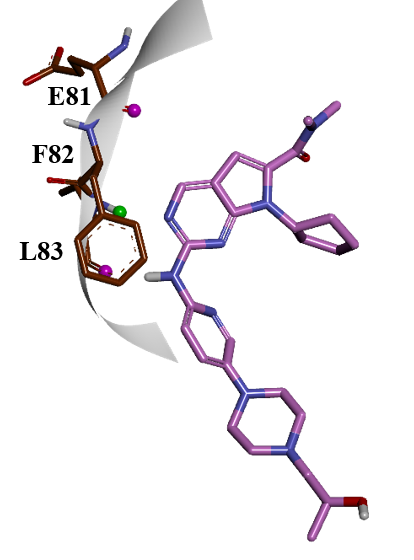 |  | 73.53 | 0.61 | 0.27 |
| ChEMBL  3658076 | 15,000 | 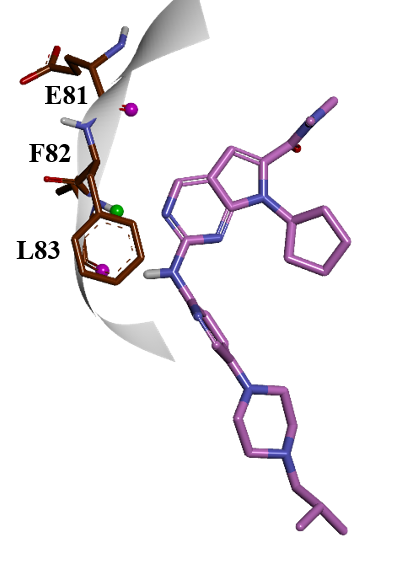 |  | 73.54 | 0.62 | 0.20 |
| ChEMBL  3658069 | 15,000 | 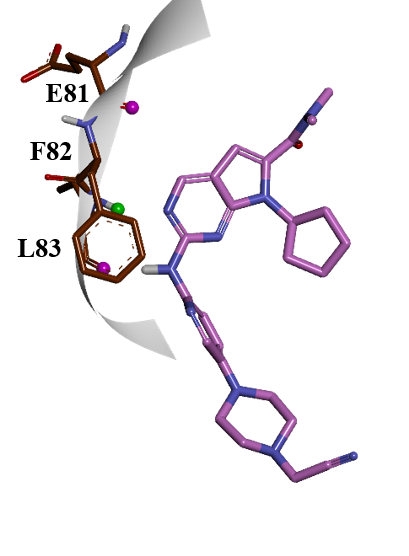 |  | 75.44 | 0.61 | 0.20 |
| ChEMBL  3654235 | 15,000 | 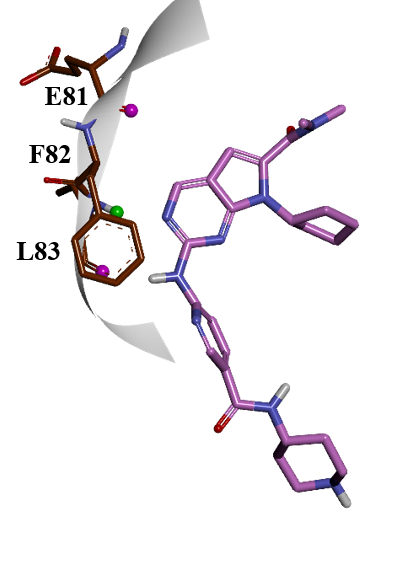 |  | 75.55 | 0.62 | 0.25 |
| ChEMBL  3658039 | 15,000 | 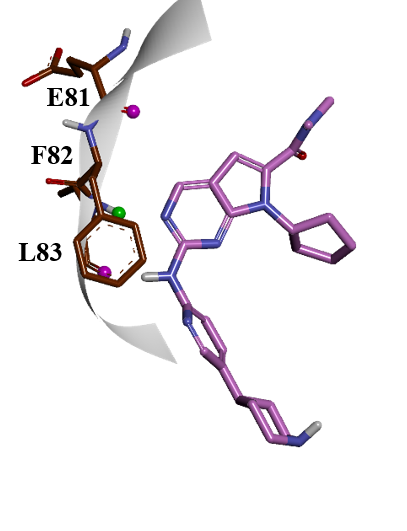 |  | 75.57 | 0.61 | 0.30 |
| ChEMBL  3639531 | 15,000 | 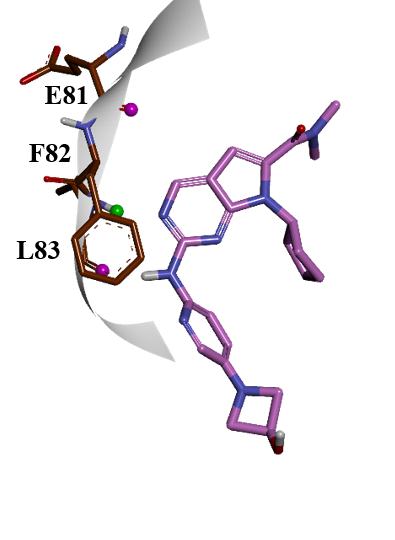 |  | 76.97 | 0.63 | 0.25 |
| ChEMBL  3658038 | 15,000 | 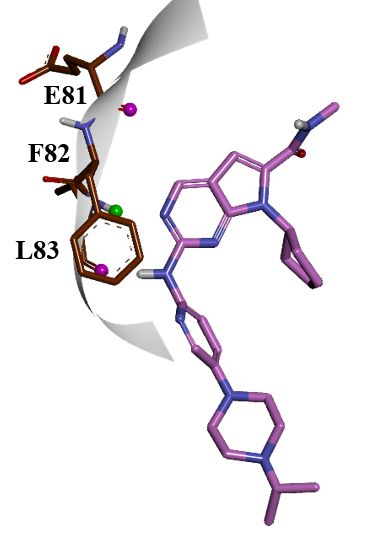 |  | 78.97 | 0.60 | 0.27 |
| ChEMBL  1222565 | 95,000 | 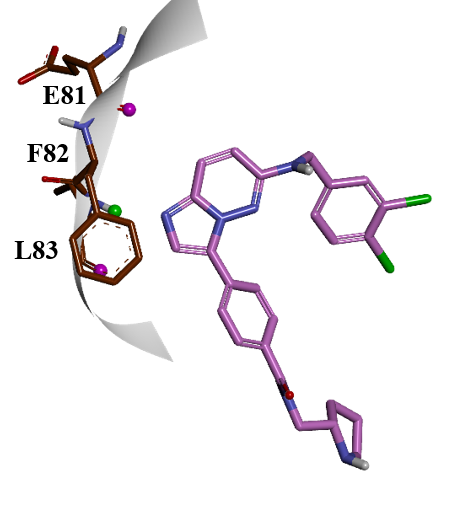 |  | 82.59 | 0.60 | 0.35 |
| ChEMBL  1222992 | 100,000 | 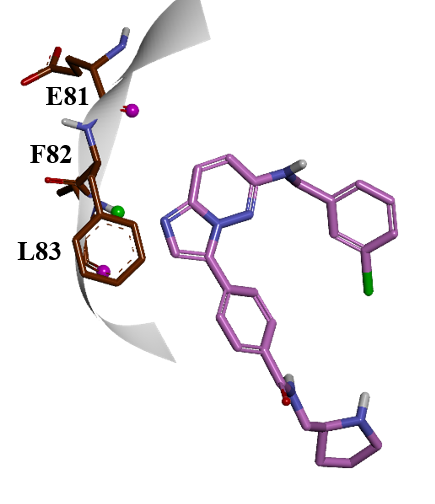 |  | 83.32 | 0.62 | 0.38 |

The * symbol in fragment structure indicates the linking part.
